# Cutaneous Fusariosis in a Pediatric Patient With Leukemia: A Diagnostic and Therapeutic Challenge

**DOI:** 10.7759/cureus.101470

**Published:** 2026-01-13

**Authors:** María T Berumen Murra, Verónica Martínez García, Guadalupe Maldonado Colín, Lucía Achell Nava, Aaron Espinosa Atri

**Affiliations:** 1 Department of Dermatology, National Medical Center 20 de Noviembre Institute of Security and Social Services for State Workers (ISSSTE), Mexico City, MEX; 2 Department of Infectious Diseases, National Medical Center 20 de Noviembre Institute of Security and Social Services for State Workers (ISSSTE), Mexico City, MEX

**Keywords:** cutaneous diseases, fusarium, immunocompromised host, mycoses, opportunistic infections

## Abstract

Fusariosis is a rare opportunistic fungal infection that primarily affects immunocompromised patients. Due to its nonspecific initial clinical manifestations, diagnosis is often delayed, which has a negative impact on patient outcomes. We report the case of an eight-year-old male patient with relapsed acute B-cell lymphoblastic leukemia and central nervous system involvement who developed fever and neutropenia during chemotherapy. Even though treatment with broad-spectrum antibiotic therapy was initiated, the patient developed disseminated cutaneous lesions characterized by erythematous macules that later progressed to necrotic plaques. Antifungal therapy with amphotericin B and voriconazole was established, but the patient’s clinical condition deteriorated rapidly, resulting in septic shock and a fatal outcome. Histopathological examination and fungal cultures confirmed the diagnosis of invasive fusariosis. This case highlights the importance of early recognition of cutaneous manifestations of fusariosis in immunocompromised pediatric patients and emphasizes the need for multidisciplinary management to improve clinical outcomes.

## Introduction

The incidence of invasive fungal infections has increased in patients with compromised immunity, reflecting both their susceptibility and the extended survival due to modern medical care [1]. Fungal infections can range from mild, superficial skin conditions to severe, life-threatening systemic illnesses. The clinical presentation varies widely depending on the patient’s immune status and overall health [1,2].

*Fusarium* species, filamentous molds with worldwide distribution, are among the pathogens capable of causing invasive disease, particularly in patients with hematologic malignancies, those receiving cytotoxic or corticosteroid therapy, and other immunocompromised populations [3,4]. These fungi can involve multiple organ systems, including the skin, lungs, and central nervous system. Cutaneous lesions occur in nearly three-quarters of disseminated cases, and the presence of multiple, painful necrotic lesions spread over the body is highly suggestive of fusariosis. However, early diagnosis remains challenging due to nonspecific clinical manifestations, overlap with other invasive mold infections, and limitations of rapid diagnostic tools [4,5].

Definitive diagnosis requires identification of the fungus [2]. Managing fusariosis is particularly difficult in clinical practice due to the intrinsic resistance of *Fusarium* species to most antifungal agents commonly used for severe fungal infections. Voriconazole and amphotericin B are frequently reported as the most effective agents in case reports [2,4,6]. Reporting this clinical case aims to highlight the diagnostic challenges, characteristic clinical features, and aggressive course of disseminated fusariosis in a profoundly immunocompromised pediatric patient, emphasizing the importance of early recognition and timely initiation of appropriate antifungal therapy.

## Case presentation

An eight-year-old male patient with a history of acute B-cell lymphoblastic leukemia, with relapse to the bone marrow and central nervous system infiltration, was treated with chemotherapy and radiotherapy. The patient presented with fever, and laboratory studies revealed an absolute neutrophil count of 50 cells/mm^3^, consistent with profound neutropenia. Empirical treatment with cefepime was initiated, with initial clinical improvement.

He remained afebrile for one week; however, he subsequently developed recurrent fever spikes. Cefepime was discontinued, and treatment with meropenem and vancomycin was initiated. Despite therapy, the fever persisted, reaching up to 39°C. Three days later, due to persistent fever, antifungal coverage was initiated with fluconazole. After five days without clinical improvement, fluconazole was replaced with caspofungin. Two days later, meropenem and vancomycin were discontinued, and linezolid and levofloxacin were started, while caspofungin was continued.

**Figure 1:**
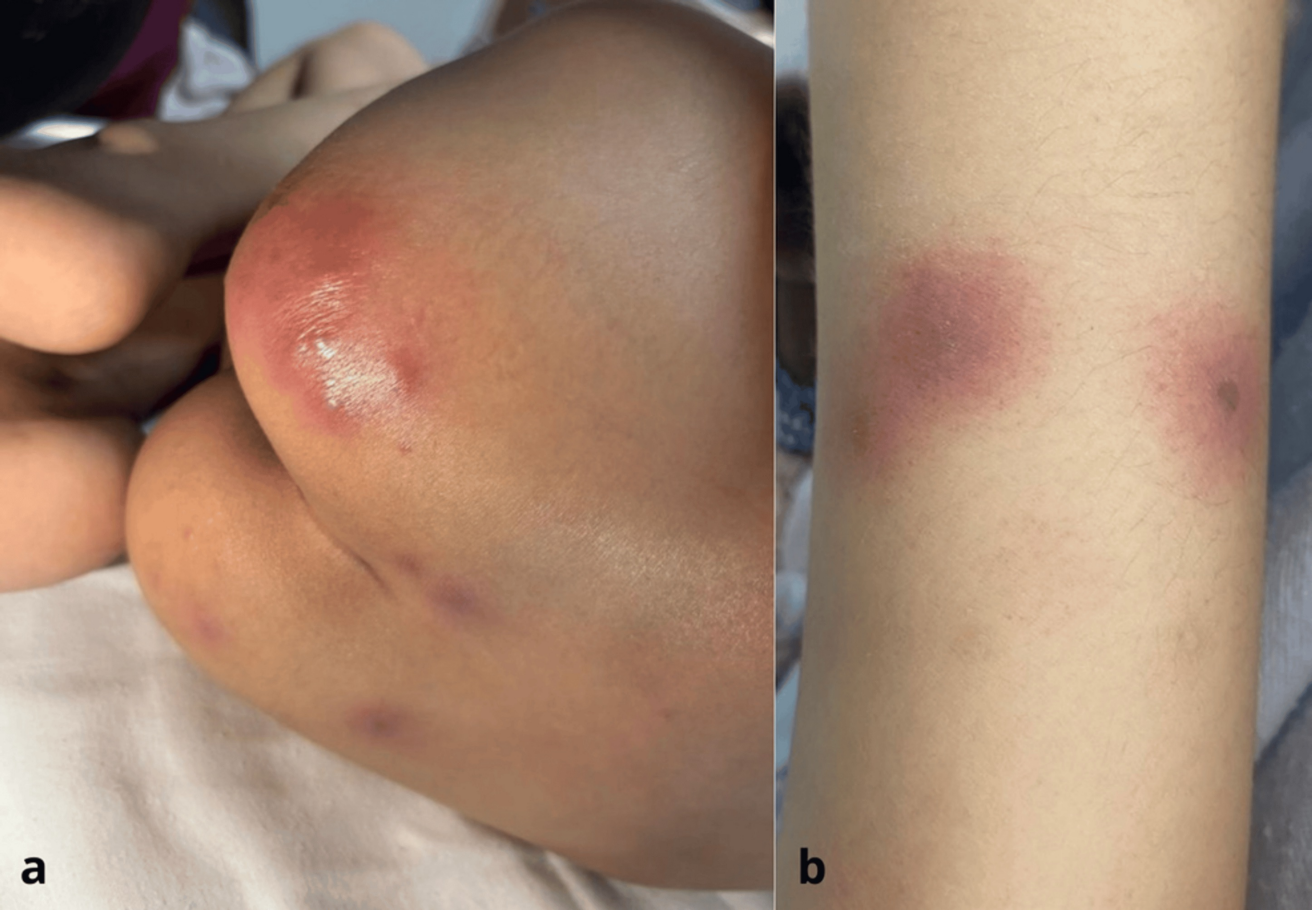
(a, b) Dermatosis on the gluteal region and right upper extremity characterized by multiple erythematous-edematous plaques with a violaceous center, circular in shape, 0.5-1 cm in diameter, with well-defined borders.

Four days later, the patient developed a disseminated dermatosis, as shown in Figures 1a, 1b, involving the face, anterior and posterior trunk, gluteal region, and upper and lower extremities. The lesions were characterized by multiple erythematous macules that progressed to erythematous-edematous plaques with a necrotic center, some of which had hemorrhagic crusts. The lesions were round, measuring 0.5 to 1 cm in diameter, with well-defined borders, and caused mild pruritus and pain. The main differential diagnoses considered were fusariosis, invasive aspergillosis, and ecthyma gangrenosum due to *Pseudomonas aeruginosa*. These conditions were considered given the presence of necrotic skin lesions in the setting of profound neutropenia and persistent fever despite broad-spectrum antimicrobial therapy. However, the rapid dissemination of multiple cutaneous lesions and the subsequent histopathological and microbiological findings were more consistent with disseminated fusariosis.

Given the suspicion of an invasive fungal infection, amphotericin B and voriconazole were added. A skin biopsy and fungal culture were performed 24 hours after the onset of the skin lesions. Three days later, the patient's clinical condition deteriorated rapidly, progressing to septic shock and cardiorespiratory arrest with a fatal outcome.

**Figure 2:**
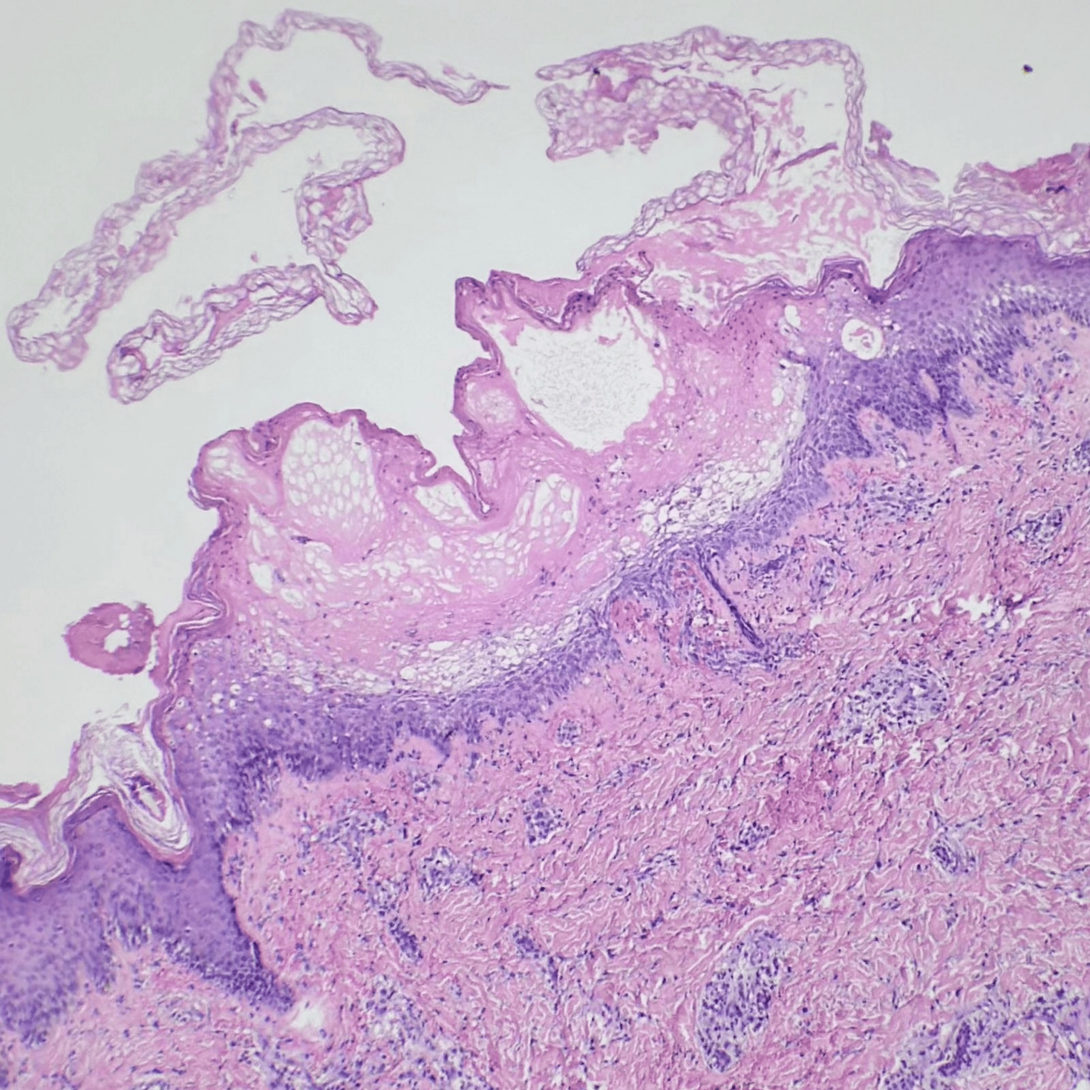
Histopathological study shows subcorneal blisters, areas of parakeratosis, and mild lymphocytic infiltrate at dermis.

**Figure 3:**
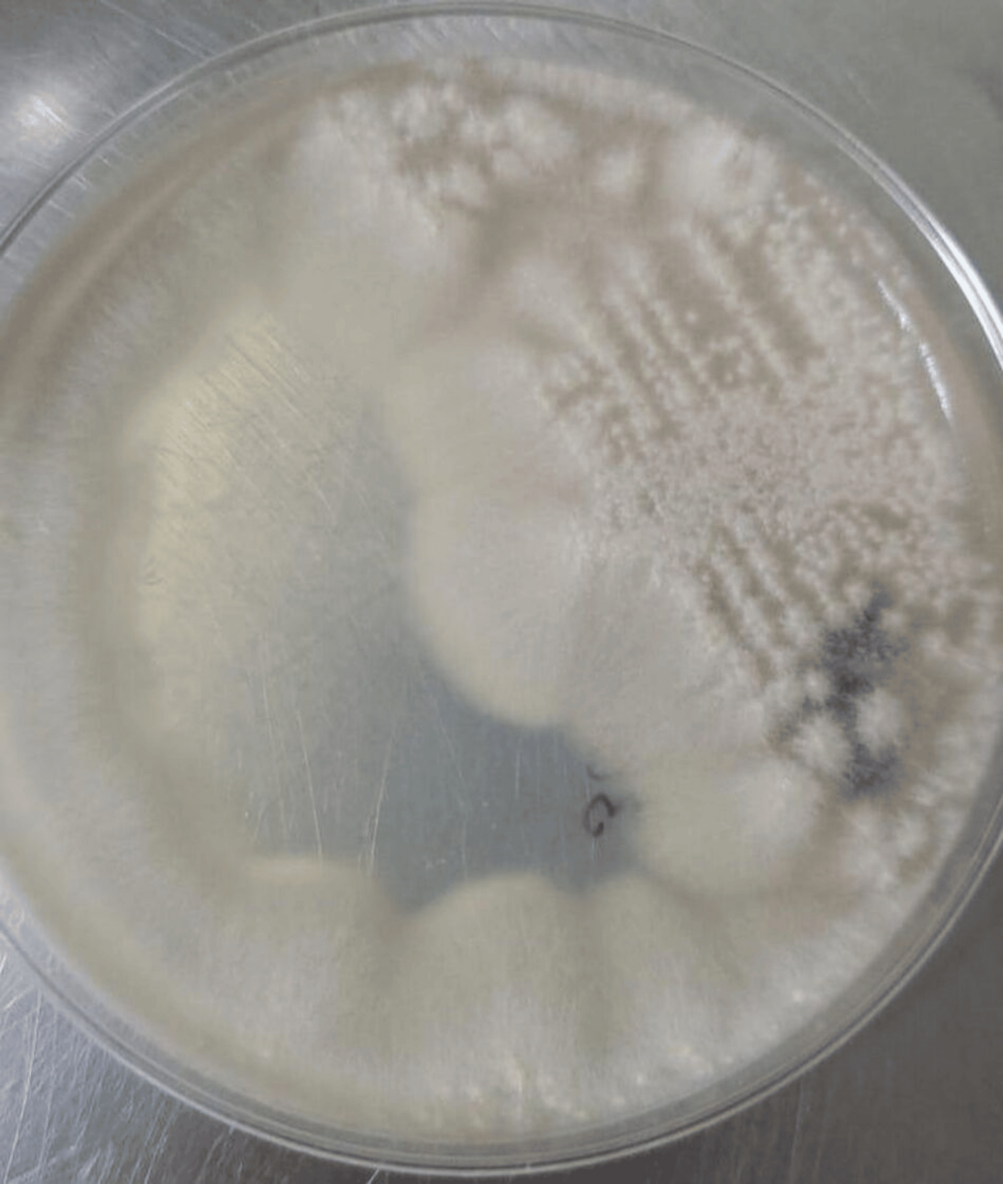
Fungal culture from the skin biopsy showed whitish, cottony colonies.

Histopathological examination of the skin biopsy, as shown in Figure 2, revealed subcorneal blisters, areas of parakeratosis, and mild lymphocytic infiltrate in the dermis. Fungal culture from the skin biopsy showed whitish, cottony colonies, as shown in Figure 3.

**Figure 4:**
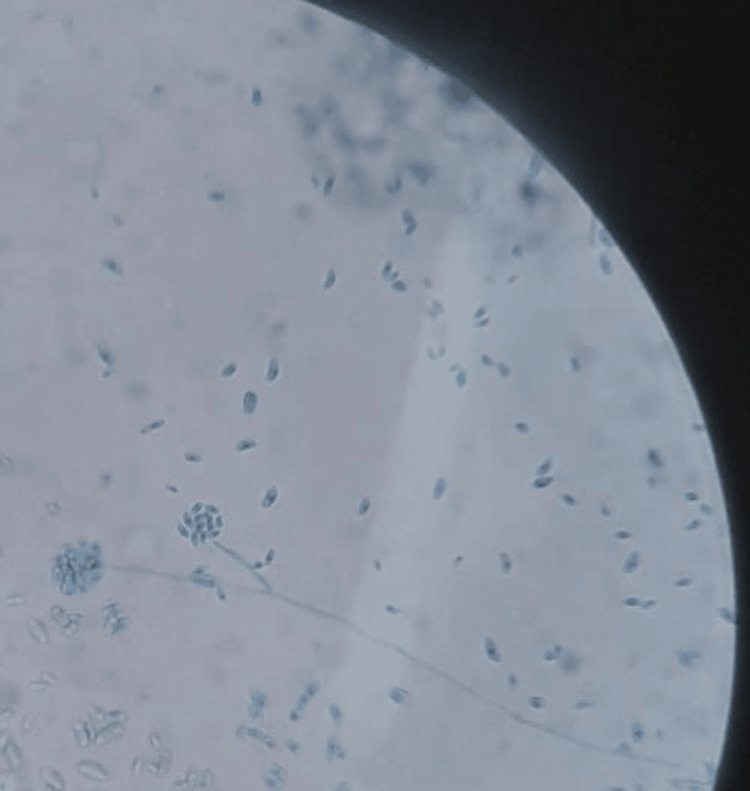
Lactophenol blue staining reveals canoe-shaped macroconidia.

Direct examination with lactophenol blue staining demonstrated canoe-shaped macroconidia, as shown in Figure 4.

**Figure 5:**
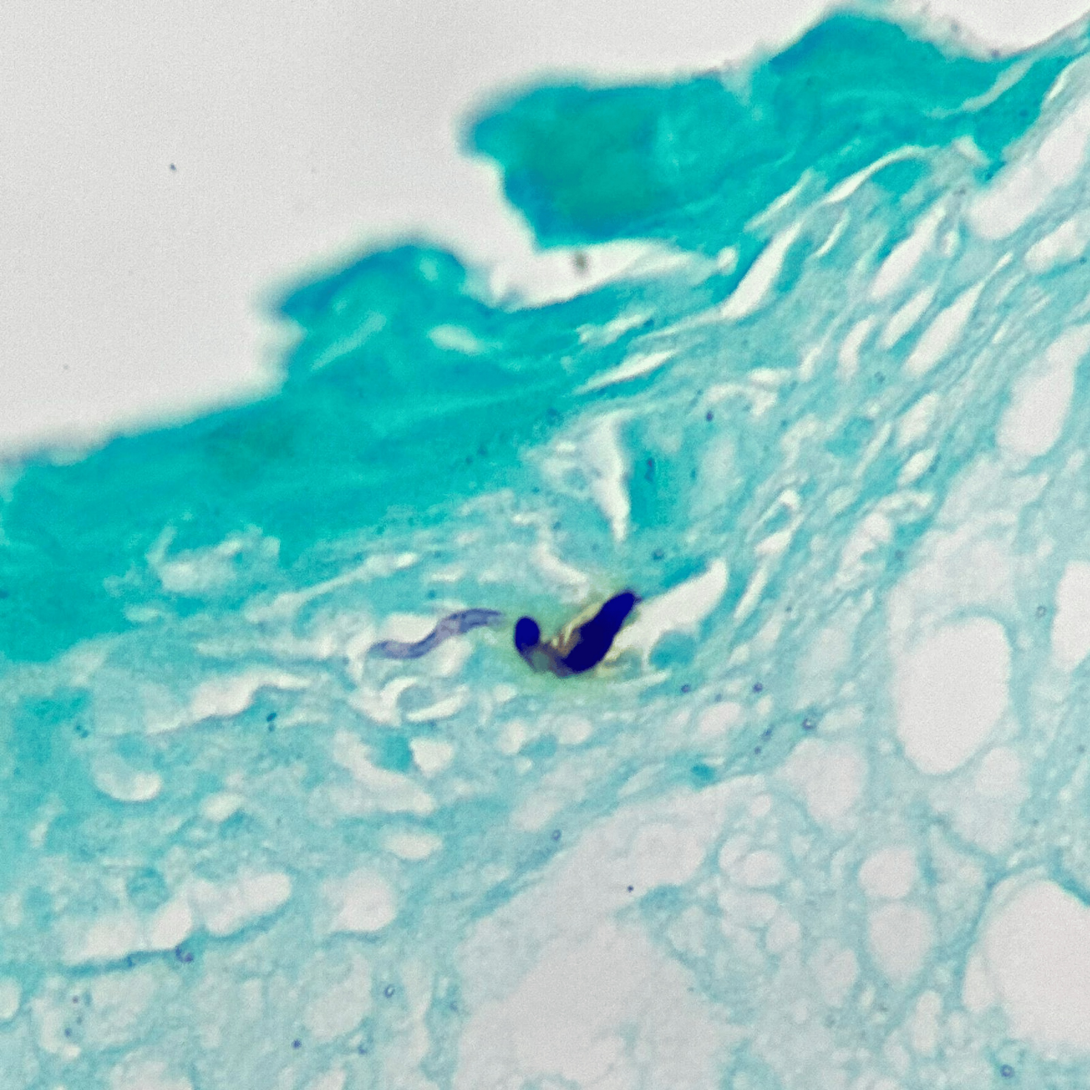
Hyaline, septate hyphae are observed with Gomori-Grocott staining.

Hyaline, septate hyphae were observed with Gomori-Grocott staining, as shown in Figure 5.

These findings confirmed the diagnosis of disseminated fusariosis. Antifungal susceptibility testing was not available at our institution.

## Discussion

*Fusarium* species are opportunistic fungi that can cause disease in humans, plants, and animals. They are filamentous, hyaline, and septate molds that can be found in soil, water, and decaying organic matter [2,5]. Among the *Fusarium* species identified in human infections, *Fusarium solani*, *Fusarium oxysporum*, *Fusarium verticillioides*, and *Fusarium proliferatum* are the most frequently reported [3,5].

The main route of infection is the respiratory tract, but it can also occur through the gastrointestinal tract, skin wounds, or devices such as catheters [3,5]. These infections present a wide clinical spectrum, which depends on the host's immune status and the route of entry [2,4]. In immunocompetent individuals, the most common clinical manifestations include onychomycosis or keratitis [4,7].

Invasive fusariosis occurs mainly in immunocompromised patients, such as those with neoplastic diseases, those receiving cytotoxic or corticosteroid therapy, individuals with diabetes, and people living with human immunodeficiency virus infection. It has been associated with hematological malignancies in 87% of cases [3]. The prevalence of invasive infection is low, estimated between 0.06% and 0.13% among patients with hematological malignancies [2].

In disseminated disease, any organ system can be affected. Skin lesions are present in 73% of patients and are characterized by painful, erythematous papules or nodules that rapidly progress to central necrosis, resembling target lesions. The lesions may be located on the face, trunk, or extremities [4,5].

In immunocompromised patients with persistent fever and necrotic cutaneous lesions, the differential diagnosis includes other invasive mold infections such as invasive aspergillosis and mucormycosis, as well as bacterial etiologies including ecthyma gangrenosum caused by *P. aeruginosa*. Although these entities may present with overlapping clinical and histopathological features, disseminated fusariosis is more frequently associated with multiple simultaneous skin lesions and a higher rate of positive blood or skin cultures. The identification of characteristic canoe-shaped macroconidia and the growth of *Fusarium* species in tissue culture were key elements in establishing the final diagnosis in this case [8,9].

Definitive diagnosis requires identification of the fungus. With Gomori's methenamine silver stain, it appears as septate hyphae with acute-angle branching. On lactophenol blue staining, macroconidia with a canoe or banana-shaped appearance are pathognomonic [2]. In addition to histopathology and tissue culture, other diagnostic modalities may aid in early detection, including blood cultures, which are positive in 80% of cases, as well as imaging studies to assess organ involvement and molecular techniques such as polymerase chain reaction or matrix-assisted laser desorption/ionization-time-of-flight (MALDI-TOF) mass spectrometry when available [10,11].

Due to the aggressive course of the infection and the high mortality rates reported in cases of fungemia from 50% to 80%, early and aggressive treatment is crucial [2,5]. *Fusarium* exhibits in vitro resistance to most of the antifungal agents commonly used to treat different types of mycosis, complicating treatment decisions [4]. The preferred agents for invasive fusariosis are voriconazole and amphotericin B; however, their efficacy is not fully established, and controversy persists as to whether they should be administered as combination therapy versus monotherapy [3,5,6]. Combination therapy using both agents, with subsequent de-escalation to monotherapy, is regarded as standard practice in certain centers. Isavuconazole has been used in the treatment of fusariosis; however, there is insufficient evidence to support recommendations [12].

Importantly, clinical outcomes in invasive fusariosis appear to be strongly influenced by host immune recovery, particularly neutrophil recovery, rather than antifungal therapy alone. Persistent neutropenia has been consistently associated with poor prognosis, whereas recovery of neutrophil counts correlates with improved survival [5,11]. In refractory or progressive cases, salvage therapeutic strategies may be considered, including combination antifungal therapy, adjustment of immunosuppression, or administration of granulocyte colony-stimulating factor, although evidence supporting these approaches remains limited [11].

## Conclusions

Fusariosis has significant diagnostic and therapeutic challenges due to its nonspecific early manifestations, rapid clinical deterioration, and limited susceptibility to many available antifungal agents. The existing therapeutic options are informed by case reports. Early identification of skin lesions and initiating antifungal treatment as soon as possible may significantly improve clinical outcomes, especially in immunocompromised pediatric patients. A multidisciplinary approach involving dermatology, infectious diseases, and hematology specialists is essential for early diagnosis and treatment. This case highlights the need to keep invasive fungal infections in mind when children with hematologic malignancies present with suggestive clinical signs.
